# Effects of surgical and FFP2 masks on cardiopulmonary exercise capacity in patients with heart failure

**DOI:** 10.1371/journal.pone.0269470

**Published:** 2022-08-23

**Authors:** Alexander Kogel, Pierre Hepp, Tina Stegmann, Adrienn Tünnemann-Tarr, Roberto Falz, Patrick Fischer, Felix Mahfoud, Ulrich Laufs, Sven Fikenzer

**Affiliations:** 1 Klinik und Poliklinik für Kardiologie, Universitätsklinikum Leipzig, Leipzig, Germany; 2 Klinik und Poliklinik für Orthopädie, Unfallchirurgie und Plastische Chirurgie, Universitätsklinikum Leipzig, Leipzig, Germany; 3 Institute of Sport Medicine and Prevention, University of Leipzig, Leipzig, Germany; 4 Klinik für Innere Medizin III, Universitätsklinikum des Saarlandes, Saarland University, Homburg (Saar), Germany; Cardiac Surgery Unit, Papardo Hospital, ITALY

## Abstract

**Aims:**

Surgical and FFP2 masks are recommended to reduce transmission of SARS-CoV-2. The cardiopulmonary effects of facemasks in patients with chronic heart failure are unknown. This prospective, cross-over study quantified the effects of wearing no mask (nm), surgical mask (sm), and FFP2 mask (ffpm) in patients with stable heart failure.

**Methods:**

12 patients with clinically stable chronic heart failure (HF) (age 63.8±12 years, left ventricular ejection fraction (LVEF) 43.8±11%, NTProBNP 573±567 pg/ml) underwent spiroergometry with and without masks in a randomized sequence. Comfort/discomfort was assessed using a standardized questionnaire.

**Results:**

Maximum power was reduced with both types of masks (nm: 108.3 W vs. sm: 101.2 W vs. ffpm: 95.6 W, p<0.01). Maximum respiratory oxygen uptake (1499ml/min vs. 1481 ml/min vs. 1300 ml/min, p = 0.95 and <0.01), peak ventilation (62.1 l/min vs. 56.4 l/min vs. 50.3 l/min, p = 0.15 and p<0.05) and O2-pulse (11.6 ml/beat vs. 11.8 ml/beat vs. 10.6 ml/beat, p = 0.87 and p<0.01) were significantly changed with ffpm but not sm. Discomfort was moderately but significantly increased (nm: 1.6 vs. sm: 3.4 vs. ffpm: 4.4, p<0.05).

**Conclusion:**

Both surgical and FFP masks reduce exercise capacity in heart failure patients, while FFP2 masks reduce oxygen uptake and peak ventilation. This reduction in cardiopulmonary performance should be considered in heart failure patients whose daily life activities are often just as challenging as exercise is for healthy adults.

## Introduction

During the ongoing COVID-19 pandemic, face masks as protective measures proved effective in decreasing the transmission of SARS-CoV-2 [1, 2]. The protection provided by FFP2 face masks is superior to surgical face masks [3]. However, concerns were raised about the ability to communicate while wearing a face mask and the impact on elderly and frail patients [4]. While data for healthy adults and the general impact of face masks are available, evidence for patients with chronic diseases and cardiovascular diseases is sparse [5–9].

Healthy adults rarely reach high levels of activity during daily activities that would incite exertion [10]. In contrast, patients with chronic heart failure (HF) reach maximum load and exertion more often in their daily lives, and they frequently adapt by decreasing intensity and prolonging the effort [11]. Hence, it is important to assess the potential effects of different face masks in these patients.

This study aims to quantitate the effects of wearing no mask (nm), surgical mask (sm), and FFP2 mask (ffpm) in clinically stable patients with chronic heart failure (HF) on optimal medical therapy. We measured well-established parameters of myocardial and pulmonary function by spiroergometry [12–15].

## Methods

### Subjects

Twelve male patients with chronic HF treated at the outpatient clinic at Leipzig University hospital participated in the study. In this study, patients with a documented diagnosis of HF with reduced or preserved ejection fraction and at least one episode of cardiac decompensation required hospitalization prior randomization were included. All patients were in a compensated status and on pharmacological therapy according to the guidelines for the medical treatment of chronic HF. The study was conducted in accordance with the latest revision of the Declaration of Helsinki. It was approved by the Ethical Committee of the Medical Faculty, University of Leipzig (reference number 328/20-ek). Written informed consent was obtained from all participants.

### Inclusion and exclusion criteria

#### Inclusion

- Clinically stable chronic heart failure
- Heart Failure with reduced Ejection Fraction (HFrEF), mildly reduced Ejection Fraction (HFmrEF), and Heart Failure with preserved Ejection Fraction, HFpEF

#### Exclusion

- Contraindications to ergometry
- Acute coronary syndrome
- Symptomatic high-grade valvular ventricular disease
- Decompensated heart failure
- Acute pulmonary embolism
- Acute inflammatory heart disease
- Acute aortic dissection
- Blood pressure at rest >180/100 mmHg
- Acute leg vein thrombosis
- Acute severe general illness
- Extracardiac disease with significantly limited life expectancy (≤6 months)
- Untreated severe ventricular arrhythmias
- Symptomatic bradycardia, AV block II° type 2 Mobitz, or AV block III° without pacemaker care
- Limited mobility with the need for walkers, wheelchair, or motorized devices without the ability to perform ergometry
- Implanted pacemaker or CRT systems (ICD allowed)
- COPD stage III

### Study design

Medical history was taken using a questionnaire. Subjects received a physical examination and documentation of vital parameters, body measurements, and a resting electrocardiogram (ECG). Each subject performed three incremental exertion tests (IET), one "no-mask" (nm), one with surgical mask (sm), and one with FFP2 mask (ffpm). The order of the testing was randomly assigned using the GraphPad Quickcalcs online randomization tool [16]. Tests were performed at the same time of day with a minimum of 48 hours between two tests. To assess baseline respiratory function, spirometry for each setting (nm, sm, ffpm) was performed. The participants were blinded about their individual test results to avoid influence by an anticipation bias.

### Incremental cardiopulmonary exertion test (CPET)

CPET were performed on a semi-recumbent ergometer (GE eBike, GE Healthcare GmbH, Solingen, Germany, Germany) at a constant speed of 55–65 revolutions per minute (rpm). Each test started with a workload of 20 W with an increase of 8 W within 1 minute (as a ramp) until voluntary exhaustion occurred. Each subject continued for an additional 5-min recovery period at a workload of 25 W.

### Masks

We used typical and widely used disposable FFP2 protective face masks (GuardweFFP2NR, Wuhan Zonsen Medial Products Co., Ltd., Wuhan City, China) and surgical masks (Suavel® Protec Plus, Meditrade, Kiefersfelden, Germany), both with ear loops. The spirometry mask was placed over the masks and fixed with head straps in a leak-proof manner as described earlier [5]. Before every run, we tested for leakage.

### Measurements

Heart rate (HR) (GE-Cardiosoft, GE Healthcare GmbH, Solingen, Germany), maximum oxygen consumption (*V*O_2max_) and minute ventilation (VE) were monitored continuously at rest, during CPET and recovery. Lung function and spirometry data were collected through a digital spirometer (Vyntus™ CPX, Vyaire Germany, Hoechberg, Germany). For each modality, (nm, sm, ffpm) data of three expiratory maneuvers with 1‐minute intervals were collected using the best values obtained for maximum forced vital capacity (FVC), forced expiratory volume in 1st second (FEV1), peak expiratory flow (PEF) and Tiffeneau index (TIFF). Capillary blood samples (55 μl) were taken from the earlobe at baseline and immediately after cessation of maximum load and analyzed by a common blood gas analyzer (ABL90 FLEX blood gas analyzer, Radiometer GmbH, Krefeld, Germany). Blood pressure (BP) was observed at rest, every 3 minutes during the CPET, and after the first 5 minutes of the recovery period.

### Quantification of comfort/discomfort

We used the questionnaire published by Li et al. to quantify the following ten domains of comfort/discomfort while wearing a mask: humidity, heat, breathing resistance, itchiness, tightness, saltiness, feeling unfit, odor, fatigue, and overall discomfort [17]. The participants were asked 10 minutes after each CPET how they perceived the comfort in the test.

### Statistical analysis

All values are expressed as means and standard deviations unless otherwise stated, and the significance level was defined as *p* < 0.05. Data were analyzed using Microsoft Office Excel^®^ 2010 for Windows (Microsoft Corporation, Redmond, Washington, USA) and GraphPad Prism 9 (GraphPad Software Inc., California, USA). For distribution analysis, the D’Agostino–Pearson normality test was used. For normal distribution, comparisons were made using one-way repeated measures ANOVA with Turkey’s post hoc test for multiple comparisons. Otherwise, the Friedman non-parametric test and Dunn’s post hoc test were used. Pearsons r was used for correlation analyses and R^2^ as the coefficient of determination. The study was powered to detect a difference of 10% in VO2max/kg between nm and ffpm with *β* = 0.2 and *α* = 0.05.

## Results

12 patients with clinically stable chronic heart failure aged 63.8±12 years, a mean left ventricular ejection fraction (LVEF) 43.8±11% and a mean NTProBNP 573±567 pg/ml were analysed. Additional patients’ baseline characteristics are depicted in **Table 1**.

**Table 1:** Baseline characteristics.

Parameter	Unit	mean ± SD		
Age	Years	63.8	±	12.6
Body mass index	kg/m^2^	31.3	±	6.1
Heart rate	Bpm	75.6	±	12.7
Systolic blood pressure	mmHg	123	±	14.5
Diastolic blood pressure	mmHg	73	±	7.8
Ejection fraction	%	43.8	±	11.2
Ischaemic heart disease	No. (%)	6		(50%)
Dilated cardiomyopathy	No (%)	4		(33%)
Chronic obstructive pulmonary disease	No (%)	2		(17%)
Glomerular filtration rate	ml/min/1.73m^2^	65	±	21.6
NT-proBNP	pg/ml	573	±	567
Beta-blockers	No. (%)	11		(92%)
ACE inhibitors	No. (%)	2		(17%)
AT1 antagonists	No. (%)	2		(17%)
Sacubitril/valsartan	No. (%)	8		(65%)
Aldosterone antagonists	No. (%)	9		(75%)
Calcium channel blockers	No. (%)	2		(17%)
Diuretics	No. (%)	8		(67%)
Lipid lowering drugs	No. (%)	9		(75%)
Antiplatelet agents	No. (%)	9		(75%)
Oral anticoagulation	No. (%)	1		(9%)
Anti-diabetic drugs	No. (%)	3		(25%)

### Spirometry

To test for effects on pulmonary function at rest we performed spirometry. The results of resting spirometry are shown in **Table 2.** Forced vital capacity was reduced by 10.2% (p<0.01) with surgical masks and by 17.2% (p<0.01) using FFP2 masks, respectively. Expiration measured as the volume that has been exhaled at the end of the first second of forced expiration was also significantly reduced by 9.3% (p<0.01) and 17.3% (p<0.01), respectively. Additionally, peak flow was slower with a reduction of 14.0% (p<0.01) and 25.1% (p<0.01).

**Table 2:** Results of the incremental cardiopulmonary exercise test.

INCREMENTAL EXERTION TEST			Unit	nm			sm			ffpm			nm vs. sm	nm vs. ffpm
Rest	Spirometry results													
	FVC	L		3.8	±	0.7	3.5	±	0.7	3.2	±	0.7	**<0.01**	**<0.01**
	FEV1	L		2.9	±	0.5	2.6	±	0.5	2.4	±	0.4	**<0.01**	**<0.01**
	PEF	l/s		6.9	±	1.8	5.9	±	1.4	5.1	±	1.6	**<0.01**	**<0.01**
	Hemodynamic parameters													
	HR	Bpm		75.6	±	12.7	77.3	±	10.6	78.3	±	13.5	ns	ns
	SBP	mmHg		123	±	14.6	115	±	14.4	112	±	18.5	ns	**<0.05**
	DBP	mmHg		73.3	±	8.7	70.8	±	6.9	72.7	±	9.3	ns	ns
	Pulmonary parameters													
	VE	l/min		11.4	±	1.9	11.1	±	2.5	11.9	±	2.9	ns	ns
	Breathing frequency	Brpm		16.1	±	4.9	13.1	±	2.5	14.4	±	3.5	ns	ns
	VT	L		0.8	±	0.3	0.9	±	0.2	0.9	±	0.4	ns	**<0.05**
	Metabolic parameters													
	pH			7.42	±	0.02	7.42	±	0.02	7.43	±	0.02	ns	ns
	PCO2	mmHg		37.2	±	3.2	37.2	±	3.7	36.2	±	4.3	ns	ns
	PO2	mmHg		72.4	±	9.4	74.1	±	13.6	74.6	±	9.1	ns	ns
Maximum	Performance													
load	Pmax	W		108.3	±	49.3	101.2	±	51.0	95.6	±	49.5	**<0.01**	**<0.01**
	Hemodynamic parameters													
	HR	Bpm		129.7	±	20.2	125.8	±	20.3	124.1	±	18.9	ns	**<0.05**
	HR/Watt	beats/W		1.35	±	0.57	1.40	±	0.54	1.48	±	0.59	ns	**<0.01**
	VO2max/kg	(ml/min)/kg		16.0	±	7.0	15.7	±	7.7	13.9	±	6.5	ns	**<0.01**
	Oxygen pulse	ml/beat		11.6	±	3.7	11.8	±	4.4	10.6	±	3.5	ns	**<0.01**
	SBP	Mmhg		176	±	40.6	169	±	32.2	165	±	37.6	ns	**<0.05**
	DBP	mmHg		79.1	±	10.3	80.1	±	13.2	77.5	±	16.7	ns	ns
	RPP (/1000)	bpm*mmhg		23.2	±	7.8	21.5	±	6.6	20.8	±	7.1	ns	**<0.05**
	RPP/Watt	bpm*mmhg/W		232.7	±	92.3	229.9	±	72.0	238.4	±	92.8	ns	ns
	Pulmonary parameters													
	VE	l/min		62.1	±	21.0	56.4	±	17.3	50.3	±	13.0	ns	**<0.05**
	Breathing frequency	Brpm		30.2	±	5.8	28.7	±	5.2	28.9	±	4.1	ns	ns
	VT	L		2.0	±	0.5	2.0	±	0.4	1.7	±	0.3	ns	**<0.01**
	Metabolic parameters													
	RER			1.08	±	0.11	1.05	±	0.08	1.05	±	0.08	ns	ns
	pH			7.36	±	0.04	7.36	±	0.05	7.36	±	0.05	ns	ns
	PCO2	mmHg		36.5	±	3.9	37.3	±	3.0	37.3	±	5.4	ns	ns
	PO2	mmHg		75.5	±	10.8	76.8	±	8.9	76.9	±	8.8	ns	ns
Overall discomfort				1.6	±	1.5	3.4	±	1.7	4.5	±	2.6	**<0.05**	**<0.01**

nm:no mask; sm: surgical mask; ffpm: FFP2 mask; FVC: forced vital capacity; FEV1: volume exhaled in the first second of forced expiration; PEF: peak flow; HR: heart rate; SBP: systolic blood pressure; DBP: diastolic blood pressure; VE: respiratory minute volume; VT: tidal volume; Pmax: maximum load achieved; RPP: rate pressure product; RER: respiratory exchange ratio.

### Incremental cardiopulmonary exercise test

**Fig 1:**
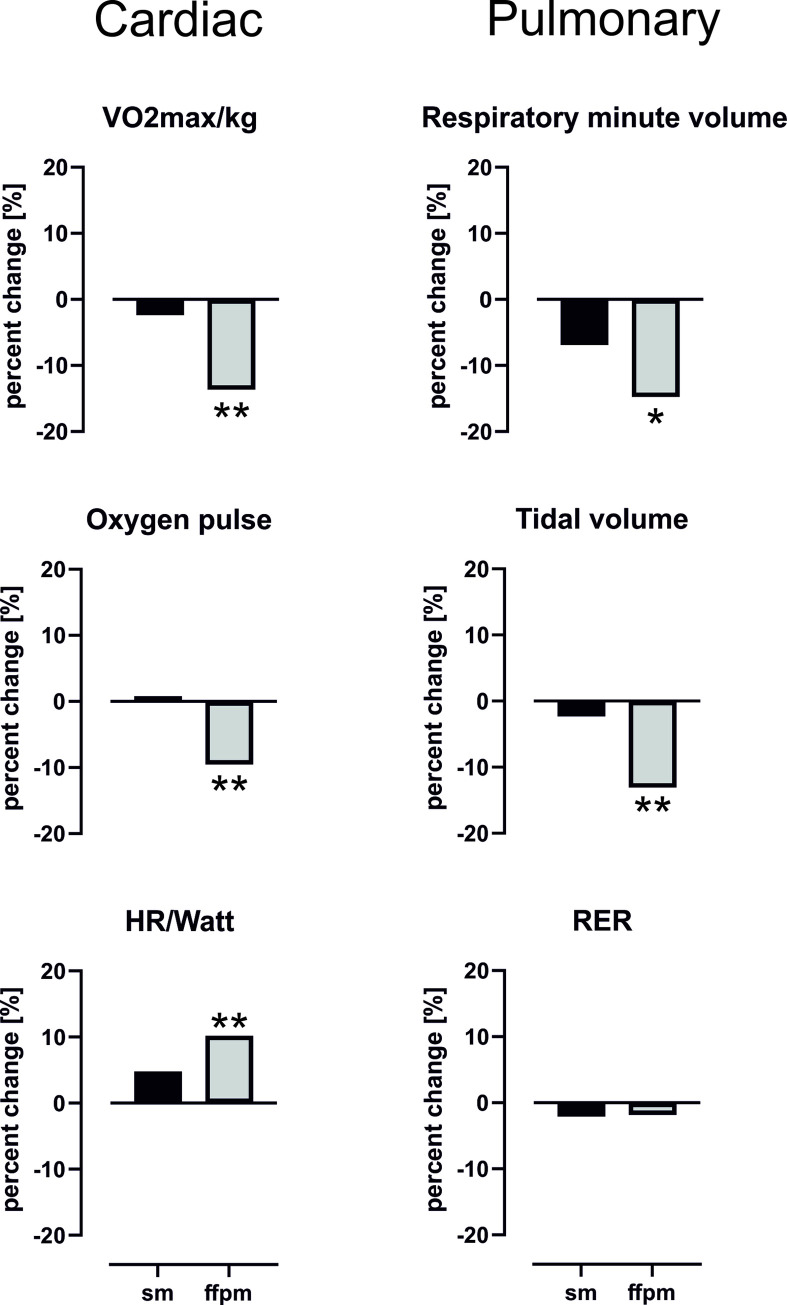
Wearing a FFP2 mask significantly changes key cardiopulmonary parameters in heart insufficient patients. VO2max: maximum respiratory oxygen uotake; HR: heart rate; RER: respiratory exchange ratio; sm: surgical mask; ffpm: FFP2 mask.

The effect of face masks on cardiopulmonary parameters under increasing loads was determined by incremental cardiopulmonary exercise test. Results and changes in parameters of the incremental cardiopulmonary exercise tests are shown in **Table 2** and **Fig 1**. Under resting conditions, the surgical mask did not affect cardiopulmonary parameters. While wearing a FFP2 mask, the tidal volume (+14.6%, p<0.05) was significantly greater than using no mask, and systolic blood pressure was significantly reduced (-8.5%, p<0.05). All other measured parameters were not significantly changed at rest.

Maximum load was significantly reduced with surgical (-7.3%, p<0.01) as well as with FFP2 masks (-12.7%, p<0.01). Surgical masks had no significant impact on cardiopulmonary parameters under maximum load. However, FFP2 masks impaired key cardiac parameters like heart rate (-4%, p<0.05), ratio between heart rate and achieved load (+10.2%, p<0.01), maximum oxygen uptake (-13.7%, p<0.01), and oxygen pulse (-9.5%, p<0.01). Additionally the systolic blood pressure (-6%, p<0.05) and the rate pressure product (-10%, p<0.05) were significantly reduced. Wearing an FFP2 mask also significantly reduced respiratory minute volume (-19%, p<0.05) and tidal volume (-15%, p<0.05). Diastolic blood pressure, breathing frequency, and metabolic parameters at maximum load did not significantly change while wearing a face mask. The significant differences measured using the FFP2 masks were associated with very high effect sizes (eta-squared) for the main endpoints: Watt (η^2^ = 0.62), VO2max (η^2^ = 0.41), and oxygen pulse (η^2^ = 0.33).

Notably, the rate pressure product relative to workload did not differ between the tests.

Pulmonary function was impacted using FFP2 masks. This resulted in a significant reduction in respiratory minute volume (-14.8%, p<0.05) and tidal volume (-13.1%, p<0.01).

### Overall discomfort

Patients reported significantly higher overall discomfort wearing masks than without. Surgical masks (+1.8 p<0.05) were reported as more comfortable than FFP2 masks (+2.9 p<0.01).

## Discussion

The main result of this study is that the physical performance of heart failure patients is impaired by face masks, especially FFP2 masks. The greatest limitations were seen in maximum respiratory oxygen uptake and respiratory volume. Nonetheless, wearing face masks does not severely lower the overall comfort of these patients while physically exercising.

### Exertion

Under all three conditions, patients reached exertion during CPET as seen by similar RER values [18]. The significantly lower heart rate while wearing a FFP2 mask could be interpreted as a sign of lower exertion levels. Notably, the ratio between heart rate and achieved load is significantly higher with FFP2 masks, an effect that would usually be expected with worsening heart failure [19]. The lower maximal heart rate is most likely an indication of a reduced ability of the failing heart to adapt. In this setup, medication with drugs reducing the heart rate is of no concern because the medication did not change between the tests, although generally beta blockade can significantly alter cardiopulmonary parameters during exercise [20]. There were no significant changes in analysed parameters between the first and the following tests, so that adequate recovery between tests can be assumed.

### Cardiac function

The systolic blood pressure decreased in the heart failure patients wearing a FFP2 mask under resting conditions. This is in contrast to findings in healthy adults [5, 21, 22]. This effect persisted through higher loads and may be a result of the heart not being able to adapt to the cardiopulmonary changes of wearing a mask. This interpretation is consistent with the significant reduction of respiratory oxygen uptake, oxygen pulse, and rate pressure product under maximum load [19]. While beta blockers can impair the increase in heart rate during exercise, they have no effect on maximum oxygen uptake [23]. Additionally, there were no changes of medication between the tests. Therefore, the changes between the tests cannot be attributed to the medication.

Comparable energy expenditure for the same load is required (RPP per Watt). Increased breathing resistance may lead to prolonged inspiration times and therefore to longer phases of higher negative intrathoracic pressure. This hypothesis is supported by the findings on inspiration times in healthy adults, which were higher while wearing a ffpm [5]. The increased cardiac preload challenges the failing heart because its limited ability to increase the stroke volume. In addition, increased transmural left ventricular pressure due to the negative intrathoracic pressure may further reduce the stroke volume [24]. The effects of breathing resistance on the cardiopulmonary system are highlighted by the use of training with breathing resistance to maximize endurance capacity and respiratory muscle function [25].

Wearing a FFP2 mask compared with no mask re-classified the heart failure patients in our study from Weber B (VO2max/kg > 16 (ml/min)/kg) to Weber C (VO2max/kg > 10–16 (ml/min)/kg) [14].

### Pulmonary function

Similar to healthy adults, the heart failure patients showed significant reductions in spirometry results while wearing a mask [5]. This effect was observed under resting and exercise conditions. Interestingly, the tidal volume was significantly increased while wearing FFP2 masks. This may be due to an anticipation of higher breathing resistance and subsequent adaptation [21]. The tidal volume and the respiratory minute volume were significantly reduced under maximum load. The higher workload for respiratory muscles compared to exercise with no mask is likely leading to exertion of these muscles and consecutively to diminished maximum pulmonary function. This again can be seen in the training effect achieved by willingly using breathing resistance to achieve higher respiratory muscle function [25]. In healthy adults, a myocardial compensation for the pulmonary limitation due to wearing a mask has been discussed [5]. Due to prolonged inspiration times, the stroke volume of the heart is increased [26]. Our data suggest that in patients with impaired myocardial function this compensation may not be possible.

### Discomfort

The participants reported a significant increase of overall discomfort from no mask over surgical masks to FFP2 masks. However, even the most discomfortable FFP2 masks were described as moderately discomfortable (4.5 of 10). Contrary, healthy adults described higher overall discomfort (7.0 of 10) under the same condition [5]. This could either be due to a habituation effect over the course of the pandemic or because heart failure patients are adapted to being restricted in their performance. Therefore, additional restrictions with masks may not cause the same level of discomfort reported by healthy persons.

### Limitations of the study

Limitations of the study include the relatively small sample size, but the study was sufficiently powered to detect a difference of 10% in VO2max/kg between nm and ffpm with *β* = 0.2 and *α* = 0.05. The study was randomized but not blinded. The external validity concerning the impact of the masks may be reduced by the laboratory conditions of wearing a spirometry mask above the tested surgical or FFP2 mask. Additionally, only one type of FFP2 mask was used. There are possible differences to the facemasks of other manufacturers. Concerns were raised about leakage during spiroergometry while wearing a face mask [27]. We tested for leakage before every run and found no indication of leakage.

There were no significant changes in analysed parameters between the first and the following tests.

## Conclusion

Wearing a face mask significantly reduces the cardiopulmonary performance of heart failure patients. Changes in critical cardiac and pulmonary parameters are more pronounced while wearing an FFP2 mask. Notably, the overall discomfort of the patients was only moderate while healthy adults described a much stronger discomfort [5]. This reduction in cardiopulmonary performance should be considered in heart failure patients for which daily life activities are often just as challenging as exercise is for healthy adults. Whether this limitation can be improved by training is not known, but it is a good target for future research.

## Supporting information

S1 Dataset (XLSX)

## References

[1] Mitze T, Kosfeld R, Rode J, Wälde K. Face masks considerably reduce COVID-19 cases in Germany. Proc Natl Acad Sci U S A. 2020; 117:32293–301. Epub 2020/12/03. doi: 10.1073/pnas.2015954117 . 33273115 PMC7768737

[2] Prather KA, Wang CC, Schooley RT. Reducing transmission of SARS-CoV-2. Science. 2020; 368:1422–4. Epub 2020/05/27. doi: 10.1126/science.abc6197 . 32461212

[3] Chu DK, Akl EA, Duda S, Solo K, Yaacoub S, Schünemann HJ. Physical distancing, face masks, and eye protection to prevent person-to-person transmission of SARS-CoV-2 and COVID-19: a systematic review and meta-analysis. Lancet. 2020; 395:1973–87. doi: 10.1016/S0140-6736(20)31142-9 . 32497510 PMC7263814

[4] Schlögl M, C A. Jones. Maintaining Our Humanity Through the Mask: Mindful Communication During COVID‐19. J Am Geriatr Soc. 2020; 68:E12–3. doi: 10.1111/jgs.16488 . 32282056 PMC7262056

[5] Fikenzer S, Uhe T, Lavall D, Rudolph U, Falz R, Busse M, et al. Effects of surgical and FFP2/N95 face masks on cardiopulmonary exercise capacity. Clin Res Cardiol. 2020:1–9. doi: 10.1007/s00392-020-01704-y . 32632523 PMC7338098

[6] Haraf RH, Faghy MA, Carlin B, Josephson RA. The Physiological Impact of Masking Is Insignificant and Should Not Preclude Routine Use During Daily Activities, Exercise, and Rehabilitation. J Cardiopulm Rehabil Prev. 2020; 41:1–5. doi: 10.1097/HCR.0000000000000577 . 33351538 PMC7769052

[7] Rebmann T, Carrico R, Wang J. Physiologic and other effects and compliance with long-term respirator use among medical intensive care unit nurses. Am J Infect Control. 2013; 41:1218–23. Epub 2013/06/12. doi: 10.1016/j.ajic.2013.02.017 . 23768438 PMC7132714

[8] Shaw K, Butcher S, Ko J, Zello GA, Chilibeck PD. Wearing of Cloth or Disposable Surgical Face Masks has no Effect on Vigorous Exercise Performance in Healthy Individuals. Int J Environ Res Public Health. 2020; 17. Epub 2020/11/03. doi: 10.3390/ijerph17218110 . 33153145 PMC7662944

[9] Epstein D, Korytny A, Isenberg Y, Marcusohn E, Zukermann R, Bishop B, et al. Return to training in the COVID-19 era: The physiological effects of face masks during exercise. Scand J Med Sci Sports. 2021; 31:70–5. Epub 2020/09/30. doi: 10.1111/sms.13832 . 32969531 PMC7646657

[10] Prince SA, Elliott CG, Scott K, Visintini S, Reed JL. Device-measured physical activity, sedentary behaviour and cardiometabolic health and fitness across occupational groups: a systematic review and meta-analysis. Int J Behav Nutr Phys Act. 2019; 16. doi: 10.1186/s12966-019-0790-9 . 30940176 PMC6444868

[11] Mapelli M, Salvioni E, Bonomi A, Gugliandolo P, Martino F de, Vignati C, et al. How Patients With Heart Failure Perform Daily Life Activities: An Innate Energy-Saving Strategy. Circ Heart Fail. 2020; 13:e007503. Epub 2020/11/17. doi: 10.1161/CIRCHEARTFAILURE.120.007503 . 33201750

[12] Gobel FL, Norstrom LA, Nelson RR, Jorgensen CR, Wang Y. The rate-pressure product as an index of myocardial oxygen consumption during exercise in patients with angina pectoris. Circulation. 1978; 57:549–56. doi: 10.1161/01.cir.57.3.549 . 624164

[13] Nelson RR, Gobel FL, Jorgensen CR, Wang K, Wang Y, Taylor HL. Hemodynamic predictors of myocardial oxygen consumption during static and dynamic exercise. Circulation. 1974; 50:1179–89. doi: 10.1161/01.cir.50.6.1179 . 4430113

[14] Weber KT, Kinasewitz GT, Janicki JS, Fishman AP. Oxygen utilization and ventilation during exercise in patients with chronic cardiac failure. Circulation. 1982; 65:1213–23. doi: 10.1161/01.cir.65.6.1213 . 6804111

[15] Wonisch M, Kraxner W, Hödl R, Watzinger N, Maier R, Hofmann P, et al. Spiroergometrie in der Kardiologie—Klinische Anwendungsmöglichkeiten. Journal für Kardiologie. 2003; 10.

[16] Randomly assign subjects to treatment groups [updated 28 Apr 2021; cited 28 Apr 2021]. Available from: https://www.graphpad.com/quickcalcs/randomize1.cfm.

[17] Li Y, Tokura H, Guo YP, Wong ASW, Wong T, Chung J, et al. Effects of wearing N95 and surgical facemasks on heart rate, thermal stress and subjective sensations. Int Arch Occup Environ Health. 2005; 78:501–9. Epub 2005/05/26. doi: 10.1007/s00420-004-0584-4 . 15918037 PMC7087880

[18] Herdy AH, Ritt LEF, Stein R, Araújo CGS de, Milani M, Meneghelo RS, et al. Cardiopulmonary Exercise Test: Background, Applicability and Interpretation. Arq Bras Cardiol. 2016; 107:467–81. doi: 10.5935/abc.20160171 . 27982272 PMC5137392

[19] Paolillo S, Agostoni P, Martino F de, Ferrazzano F, Marsico F, Gargiulo P, et al. Heart rate during exercise: mechanisms, behavior, and therapeutic and prognostic implications in heart failure patients with reduced ejection fraction. Heart Fail Rev. 2018; 23:537–45. doi: 10.1007/s10741-018-9712-1 . 29926282

[20] Fikenzer S, Fikenzer K, Laufs U, Falz R, Schulze A, Busse M. Effects of cardioselective beta-blockade on plasma catecholamines and performance during different forms of exercise. J Sports Med Phys Fitness. 2020; 60:643–9. Epub 2019/12/05. doi: 10.23736/S0022-4707.19.10225-3 . 31818057

[21] Lässing J, Falz R, Pökel C, Fikenzer S, Laufs U, Schulze A, et al. Effects of surgical face masks on cardiopulmonary parameters during steady state exercise. Sci Rep. 2020; 10:22363. Epub 2020/12/21. doi: 10.1038/s41598-020-78643-1 . 33349641 PMC7752911

[22] Chapman KR, D’Urzo AD, Druck MN, Rebuck AS. Cardiovascular response to acute airway obstruction and hypoxia. Am Rev Respir Dis. 1989; 140:1222–7. doi: 10.1164/ajrccm/140.5.1222 . 2817585

[23] Head A. Exercise metabolism and beta-blocker therapy. An update. Sports Medicine. 1999; 27:81–96. doi: 10.2165/00007256-199927020-00002 . 10091273

[24] Cheyne WS, Harper MI, Gelinas JC, Sasso JP, Eves ND. Mechanical cardiopulmonary interactions during exercise in health and disease. J Appl Physiol (1985). 2020; 128:1271–9. Epub 2020/03/12. doi: 10.1152/japplphysiol.00339.2019 . 32163324

[25] Kido S, Nakajima Y, Miyasaka T, Maeda Y, Tanaka T, Yu W, et al. Effects of Combined Training with Breathing Resistance and Sustained Physical Exertion to Improve Endurance Capacity and Respiratory Muscle Function in Healthy Young Adults. J Phys Ther Sci. 2013; 25:605–10. doi: 10.1589/jpts.25.605 . 24259812 PMC3804987

[26] Ryan KL, Cooke WH, Rickards CA, Lurie KG, Convertino VA. Breathing through an inspiratory threshold device improves stroke volume during central hypovolemia in humans. J Appl Physiol (1985). 2008; 104:1402–9. Epub 2008/02/28. doi: 10.1152/japplphysiol.00439.2007 . 18309096

[27] Shaw KA, Zello GA, Butcher SJ, Ko JB, Bertrand L, Chilibeck PD. The impact of face masks on performance and physiological outcomes during exercise: a systematic review and meta-analysis. Appl Physiol Nutr Metab. 2021; 46:693–703. Epub 2021/04/26. doi: 10.1139/apnm-2021-0143 . 33901405

